# How Can the Scope of a New Global Legally Binding Agreement on Plastic Pollution to Facilitate an Efficient Negotiation Be Clearly Defined?

**DOI:** 10.1021/acs.est.1c02033

**Published:** 2021-04-28

**Authors:** Yangzhao Sun, Yan Lin, Eirik Hovland Steindal, Chao Jiang, Sen Yang, Thorjørn Larssen

**Affiliations:** † Norwegian Institute for Water Research Headquarter, Oslo 0349, Norway; ‡ Norwegian Institute for Water Research China Office, Beijing, 100040, China

**Keywords:** plastic pollution, marine litter, microplastics, environmental agreement, life cycle management

Crossing borders both as a truly globalized commodity and eventually as unmanaged waste, plastic pollution warrants joint global action. Plastic pollution has the potential to spread toxic chemicals intentionally added to them or passively adsorbed from the environment, including persistent organic pollutants, endocrine disruptor chemicals, and heavy metals, posing enormous risks to marine ecosystems, biodiversity, and food availability.^1^ With the unprecedented concern already having been raised worldwide, the problem was further aggravated by the COVID-19 pandemic. The urgency of addressing plastic pollution was reiterated by countries at the first session of the Fifth United Nations Environment Assembly (UNEA-5.1) on February 22–23, 2021. At the meeting, delegates stressed the inadequacy of existing international legal and policy frameworks and the trans-boundary characteristics of plastics. At least 40 countries expressed support for a new global agreement on plastic pollution.^2^

In international diplomacy and policy negotiations, the quest for accuracy of wording is almost extreme. A miniscule variation in wording can lead to a huge change in scopes and responsibilities. The terms “marine plastic litter” and “microplastics” are commonly cited in the relevant discussions, policy papers, and resolutions. Other terms, such as “marine litter”, “marine debris”, and “plastic pollution”, were also used by country leaders at UNEA-5.1. The different wordings may, intentionally or not, reflect the speakers’ different focuses and objectives with regard to the plastic issue. Thus, the lack of a defined terminology may trigger confusion, misinterpretation, and resource-demanding processes. Disentangling these potential differences and defining a common objective early on may contribute to enhancing stakeholder engagement and facilitate a more streamlined negotiation process. This article will shed light on some of the more profound divergences in ongoing international deliberations and some critical intersections on the road to a negotiation mandate for a future plastic agreement.

In each of the UNEA meetings since 2014, plastic has been a key topic. Different framings of the plastic pollution issue can be observed in its resolutions and countries’ statements. The resolutions adopted were titled “Marine Plastic Debris and Microplastics” (EA.1/Res.6), “Marine Plastic Litter and Microplastics” (EA.2/Res.11), “Marine Litter and Microplastics” (EA.3/Res.7), and “Marine Plastic Litter and Microplastics” (EA.4/Res.6), the last of of which is currently the most widely accepted. At UNEA-5.1, various countries intervened on the issue using terms such as “marine plastic litter and pollution of microplastics” and “marine litter and plastic pollution”.^3^ No doubt, the marine ecosystem is the end point of large amounts of pollution and there has been significant global attention paid to the emerging environmental problems related to plastic pollution, particularly to the marine environment. However, the “marine” label may also depict a boundary for action, essentially relegating the solution to the end of the pipe after the plastics are out in the environment and potentially limiting options for eliminating waste across the entire life cycle of plastics. With over 80% of ocean plastic coming from land-based sources, the solution of marine plastic pollution is mainly land-based and upstream. Hence, an overly narrow marine scope of the international deliberations may fail to incorporate key land-based upstream sources and corresponding mitigative measures, such as sustainable design, production, and consumption.

At this stage, if the scope of plastic pollution is solely a marine issue, this may also affect the support and involvement in a treaty process. In many countries, marine environmental protection is usually under the auspices of a specific management body dedicated to marine issues. On the other hand, the current global negotiation is facilitated by the UN Environment Programme and mainly participated by the environmental departments of each country. However, as it is predominantly a land-based issue, plastic pollution will in most countries require the involvement of various departments and bodies, including industrial development and planning, agriculture, waste management, environmental protection, and so on. A marine label could potentially be misleading in that sense.

In most of the processes addressed above, the term “microplastics” accompanies marine plastic litter. Microplastics are generally defined as plastic materials smaller than 0.5 cm in diameter, categorized as primary microplastics such as microbeads and secondary microplastics. The latter accounts for 69–81% of microplastics found in the oceans.^4^ Microplastics do not deviate from regular plastic pollution with regard to the source and management perspective; reducing microplastic pollution will mainly rely on source control and reduced mismanaged plastic waste which could be achieved together with the regular plastic pollution reduction and prevention actions. Hence, in terms of policy framework development, we argue that microplastics should be a subcomponent of plastic pollution rather than dealt with separately.

Furthermore, “litter”, “pollution”, “waste”, and “debris” have similar meanings but may lead to confusion regarding the scope and responsible parties if they are not clearly distinguished. Litter is objects strewn or scattered at an unsuitable location. Pollution indicates a negative effect on the environment, and litter may cause pollution. Waste is an unwanted substance. Waste may turn into litter and cause pollution if it is not captured by a waste management system or not properly treated. Debris is the remains of anything broken down or destroyed. If the goal of a new global agreement is to holistically address the environmental impacts of plastic throughout its life cycle, we opine that the scope of “pollution” may be more justifiable and comprehensive than that of “litter”, “debris”, and “waste”.

Looking ahead, assuming that the international community will reach consensus to develop a new legally binding agreement to address plastic pollution, the scope of the agreement will be a priority topic to be addressed. A new global agreement must consider the broader challenges that underpin the processes leading to leakage of plastics into the environment.^5^ As discussed above, terms such as “marine”, “litter”, “waste”, and “debris” may contribute to limiting the scope of the agreement, failing to address plastic’s entire life cycle and broad environmental impact. Along the same line of argumentation, microplastics should be a subordinate topic to plastic pollution. Any mandate for such negotiations should ensure that all relevant sectors and processes throughout the life cycle of plastics will be part of the negotiations. The new agreement should be a multisectoral and multistakeholder agreement with a combination of legally binding and voluntary measures to control plastic pollution.
